# Oropharyngeal dysphagia and associated factors among individuals living in nursing homes in northern Sweden in 2007 and 2013

**DOI:** 10.1186/s12877-022-03114-3

**Published:** 2022-05-13

**Authors:** Patricia Hägglund, Maria Gustafsson, Hugo Lövheim

**Affiliations:** 1 Department of Clinical Sciences, Speech-Language Pathology, Umeå University, Umeå, Sweden; 2 Department of Integrative Medical Biology, Umeå University, Umeå, Sweden; 3 Department of Community Medicine and Rehabilitation, Geriatric Medicine, Umeå University, Umeå, Sweden; 4 Wallenberg Centre for Molecular Medicine, Umeå, Sweden

**Keywords:** Swallowing disorder, Deglutition disorder, Cognitive impairment, Dementia, ADL dependency, Artificial nutrition, Malnutrition, Chewing ability, Nursing home

## Abstract

**Background:**

Swallowing difficulties in the oral cavity or pharynx (i.e., oropharyngeal dysphagia) are a common problem in the aging population, which may result in severe consequences, such as malnutrition, aspiration pneumonia, and mortality. Identifying oropharyngeal dysphagia and its associated factors is essential for establishing better healthcare policies in nursing homes. In this study, we aimed to describe the oropharyngeal dysphagia prevalence among nursing home residents, and to investigate the association between dysphagia and potentially related factors in a large survey of nursing home residents in Sweden, including individuals with various degrees of cognitive impairment. A secondary aim was to compare findings between years on oropharyngeal dysphagia and its associated factors.

**Methods:**

This study is based on two cross-sectional surveys performed in 2007 and 2013, including 4,995 individuals living in nursing homes in the Region of Västerbotten, Sweden. Data were collected from caregivers’ reports regarding swallowing ability, nutritional status, chewing ability, and other baseline characteristics, such as cognitive function and activity of daily living (ADL). Data were analyzed using logistic regression models to calculate the odds of the association between oropharyngeal dysphagia and associated factors.

**Results:**

Oropharyngeal dysphagia was reported in 14.9% (95% CI: 13.9–16.0) of the nursing home residents. An adjusted model revealed that oropharyngeal dysphagia was associated by severe cognitive impairment (OR: 1.56, 95% CI: 1.14–2.12) and ADL independence (OR: 0.81 95% CI: 1.82–2.66) among nursing home residents. We also identified the following as independently associated factors of dysphagia: reduced nutritional status (OR: 1.84, 95% CI: 1.49–2.27), artificial nutrition (OR: 6.33, 95% CI: 2.73–14.71), and clinical signs of aspiration (OR: 10.89, 95% CI: 8.40–14.12).

**Conclusions:**

Oropharyngeal dysphagia was reported among approximately 15% nursing home residents and was associated with cognitive impairment and ADL capability. Furthermore, reduced nutritional status and artificial nutrition were also associated with oropharyngeal dysphagia. Implementing routine protocols in nursing homes may help detect oropharyngeal dysphagia and manage oropharyngeal dysphagia among residents.

## Background

Oropharyngeal dysphagia refers to swallowing difficulties in the oral cavity or pharynx, and is a common problem in the aging population due to age-related diseases and changes that affect swallowing function [1]. Oropharyngeal dysphagia can result in two types of potentially severe and life-threatening complications: impaired swallowing efficacy that may cause malnutrition or dehydration, and impaired swallowing safety that may cause choking, aspiration pneumonia, and mortality [1–3]. Such swallowing difficulties are also associated with anxiety, depression, and reduced quality of life [1, 4, 5]. Common signs of oropharyngeal dysphagia include coughing and wet or gurgly voice (signs of impaired swallowing safety) and drooling or food getting stuck in the throat (signs of impaired swallowing efficacy) before, during, or after swallowing. Although oropharyngeal dysphagia is a known risk factor for nursing home-acquired pneumonia [6], nursing homes often lack protocols for diagnosing and routinely screening for swallowing problems [7]. Identifying and managing oropharyngeal dysphagia and its associated factors are essential for establishing better healthcare policies for the aging population in nursing homes.

In recent years, oropharyngeal dysphagia has been recognized as a geriatric syndrome [1], as it is multifactorial and related to neurogenic and neurodegenerative processes, muscular weakness, and sarcopenia [8]. Furthermore, it is associated with poor outcomes, such as increased dependency and mortality [3, 9], and is highly prevalent in old age [1, 8, 10]. Oropharyngeal dysphagia risk factors include age-related changes of the swallowing process (known as prespyphagia), along with the increased prevalence of age-related diseases that cause pathophysiological alterations affecting the swallowing process (e.g., stroke, Parkinson’s disease, and major neurocognitive disorder) [1].

Oropharyngeal dysphagia is a significant healthcare problem among nursing home residents, with a reported prevalence ranging from 12% to 52.7% [11–14]. The oropharyngeal dysphagia prevalence rates in the literature vary as a result of participant selection (e.g., whether people with severe cognitive impairment are included), screening or assessment tools (e.g., instrumental assessment, such as videoendoscopy, results in higher detection), and the utilized definition of dysphagia [1]. Oropharyngeal dysphagia rates range from 37–78% among patients with stroke, and from 19–84% in patients with major neurocognitive disorder, with the prevalence depending on the stage and type of neurocognitive disorder and assessment tool used [1]. Despite the rising number of older people with cognitive impairment suffering from oropharyngeal dysphagia among nursing home nursing home residents, few studies have investigated its prevalence and associated factors in this high-risk population [11, 13]. The studies that have investigated dysphagia among nursing home residents, have reported that increased oropharyngeal dysphagia risk is associated with moderate to severe neurocognitive disorder [11, 12], severe functional dependency [11, 14], neurologic diseases [12], polymedication [12], chewing problems [12], malnutrition [11, 12], and tube feeding [12, 14]. Higher oropharyngeal dysphagia risk has also been observed among nursing home residents with reduced oral intake [12], and swallowing ability is reportedly related to dental status [15].

Understanding the prevalence of and factors associated with oropharyngeal dysphagia among nursing home residents can help in establishing better healthcare policies and improved oropharyngeal dysphagia management. In the present study, we aimed to describe the prevalence of oropharyngeal dysphagia among nursing home residents, and to investigate the association between oropharyngeal dysphagia and potentially related factors in a large survey of nursing home residents in Sweden performed in 2007 and 2013, including individuals with various degrees of cognitive impairment. A secondary aim was to compare findings between years on oropharyngeal dysphagia and its associated factors.

## Methods

### Study design

This study is a cross-sectional study based on two questionnaire surveys conducted in 2007 and 2013. These surveys constitute part of a recurrent survey of nursing home residents, conducted every six or seven years since 1975 [16–18]. This recurrent survey aims to describe the population living in a nursing home in the Region of Västerbotten regarding cognition, function, behavioral and psychological symptoms, and other health-related factors, including swallowing. This study was carried out in accordance with the Helsinki declaration and was approved by the Regional Ethical Review Board in Umeå, Sweden (Dnr 07-028 M and 2012–646-31 M).

### Participants and settings

**Fig. 1:**
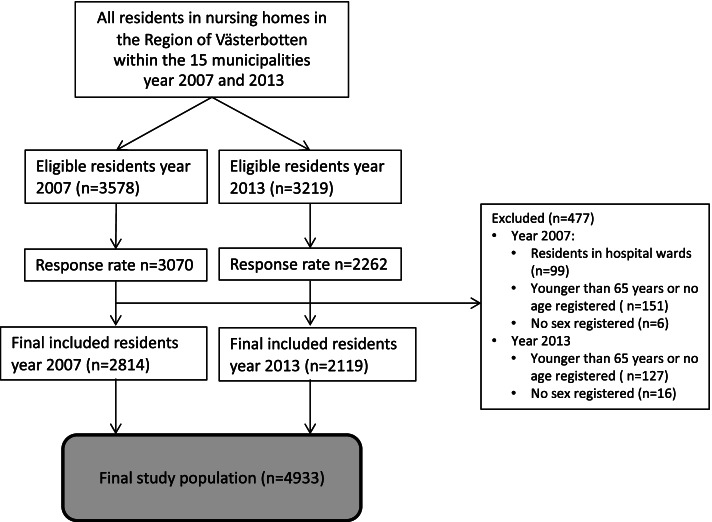
Flow-chart of the inclusion process

All individuals aged 65 years or older, living in nursing homes within the 15 municipalities in the Region of Västerbotten in northern Sweden, including specialized care units for people with major neurocognitive disorder, were eligible to participate in the present study. A total of 3,578 people were eligible in 2007, and 3,210 in 2013. The response rates were 85.8% (*n* = 3,070) in 2007, and 70.5% (*n* = 2,262) in 2013. The survey also included people in geriatric and psychogeriatric hospital wards in 2007, but not in 2013. Therefore, in our present study, we excluded people in hospital wards (99 people in 2007) from the data analyses. People were also excluded from the data analyses if they were younger than 65 years or had no age registered (151 people in 2007, and 127 people in 2013), or had no sex registered (6 people in 2007, and 16 people in 2013). Therefore, the final sample included 4,933 people (2,814 people in 2007, and 2,119 in 2013), see flow-chart (Fig. 1). Table 1 shows the basic characteristics of the residents*.*

**Table 1:** Participants’ characteristics in nursing homes and distribution of results between year 2007 and 2013

	Total *n* = 4933	2007 *n* = 2814	2013 *n* = 2119	*P* value
Mean age ± SD	84.7 ± 6.9	84.6 ± 6.8	85.0 ± 7.0	0.073^a^
Sex				0.627^b^
* Women*	3359/4933 (68.0)	1924 (68.4)	1435 (67.7)	
Cognitive score (0–27), mean ± SD	16.2 ± 8.6	16.2 ± 8.9	16.5 ± 8.4	0.235^a^
Cognitive impairment^c^	3061/4371(70.0)	1767/2531 (69.8)	1294/1840 (70.3)	0.715^b^
ADL score (4–24), mean ± SD^d^	15.5 ± 6.3	15.5 ± 6.3	15.6 ± 6.2	0.504^a^
Walks independently^e^	2519/4840 (52.0)	1324/2787 (47.5)	1195/2053 (58.2)	< 0.001^b^
Intelligible speech^f^	3637/4673 (77.8)	2062/2739 (75.2)	1575/1934 (81.4)	< 0.001^b^
Polymedication^g^	1547/4701 (32.9)	946/2814 (33.6)	601/1887 (31.8)	0.206^b^

Data are given as *n* (%) or as otherwise noted

*SD* standard deviation, *ADL* activities of daily living,

^a^Independent t-test

^b^Chi-squared test

^c^Defined as Gottfries’ score of < 24 points. Missing data on 562 residents

^d^Missing data on 240 residents

^e^Missing data on 93 residents

^f^Defined as understandable speech, i.e., individuals able to make themselves understood. Missing data on 260 residents

^g^Defined as regularly taking 5 or more different drugs. Missing data on 232 residents

### Materials

The questionnaire survey used in the present study included the Multi-Dimensional Dementia Assessment Scale (MDDAS) [16, 18]. The MDDAS contains items covering activities in daily living (ADL), behavioral and psychological symptoms, and cognitive function based on Gottfries’ cognitive scale [16, 18–20]. The survey also included questions regarding swallowing ability, nutritional status, and chewing ability.

The ADL score was based on the resident’s ability to perform the following functions: hygiene, dressing, eating, bladder control, and bowel control. All items were scored 1–5, except bladder control, which was scored 0–4. The total ADL score ranges from 4–24, where a higher score indicates greater independence in ADL.

Cognitive function was measured using a scale developed by Gottfries and Gottfries [19, 20], which comprises 27 items that measure a person’s cognitive function. A score of < 24 indicates cognitive impairment, which also correlates with a sensitivity of 90% and specificity of 91% [16] to the cut-off score of 24/30 on the Mini-Mental State Examination (MMSE) [21].

Swallowing ability was estimated based on answers of “Yes” or “No” to the following clinically relevant questions regarding symptoms of oropharyngeal dysphagia: “Does the patient have difficulty swallowing?” (i.e., dysphagia) and “Has the patient been aspirating (misdirected swallow) during food intake in the last week?” (i.e., clinical signs of aspiration). The present study thus defined oropharyngeal dysphagia as having difficulties swallowing and/or showing clinical signs of aspiration. No further explanation on the term and definition of oropharyngeal dysphagia was provided. Nutritional status was estimated based on answers of “Yes” or “No” to the following questions: “Has the patient’s nutritional intake decreased in the last three months due to loss of appetite?”, “Does the patient have a percutaneous endoscopic gastrostomy (PEG) feeding tube?”, and “Does the patient have a nasogastric feeding tube?”. The residents’ chewing ability was also estimated as good, quite good, or poor.

### Procedure/data collection

The surveys were sent to the nursing home settings, with written instructions on how to complete each assessment form based on observations during one week. The form was filled out by the caregivers (nursing staff) who knew the resident best, which was decided within each working group in the nursing homes, i.e., a proxy method was used for the data collection. The caregivers were not further trained in the data assessment but could contact members of the research group by telephone at any time, if needed. After completion, the forms were sent back to the research group.

### Statistics

Descriptive data are presented as number (%) for categorical variables, and as mean (± SD) for continuous variables. Comparisons were made using the Chi-square test for categorical variables, and t-test for continuous variables. Logistic regression was used to compare the oropharyngeal dysphagia prevalence between investigation years, with adjustment for cognition (Gottfries’ cognitive scale), ADL capability, age, and sex. Logistic regression was also used to calculate the odds ratio (OR) with a 95% confidence interval (CI) for the association between potential predictors and dysphagia, and between oropharyngeal dysphagia and its potential consequences (with separate models for each potential consequence). *P* < 0.05 was considered significant. All statistical analyses were performed using IBM/SPSS version 28.

For cognition (Gottfries’ cognitive scale) the prevalence of oropharyngeal dysphagia was plotted for each score, to visualize possible non-linear association. These plots guided the choice of how to include these variables in the logistic analysis of association between oropharyngeal dysphagia and potential predictors. Cognition was hence modeled using one continuous (linear) variable and one dichotomized variable (for very low cognitive function: Gottfries’ scores 0–2), to fit the non-linear association between oropharyngeal dysphagia and cognition. Therefore, cognition was represented by two variables in the final adjusted logistic regression models. Regarding missing data in the analyses performed, the missing data were between 3 and 9 percent of the total study sample in different variables and was handled by not including those individuals in the specific analysis of that variable. If a resident was missing three or fewer items in the Gottfries’ cognitive scale, the sum score was imputed according to the suggested imputation strategy [19, 20].

## Results

### Participants

Table 1 presents the residents’ characteristics. The residents’ ability to walk independently and ability to make themselves understood differed between the years 2007 and 2013. No other differences were noted among the residents’ characteristics.

### Swallowing ability, nutritional status, and chewing ability between years

Table 2 presents the prevalence of swallowing ability, nutritional status, and chewing ability, as assessed by caregivers, between years. The caregivers reported oropharyngeal dysphagia among 14.9% (95% CI: 13.9–16.0) of the residents, with greater prevalence in 2007 (16.2%, 95% CI: 14.8–17.6) than in 2013 (13.2%, 95% CI: 11.8–14.8, OR_adj_: 0.96, 95% CI: 0.93–0.99, *P* = 0.004), when adjusted for age, sex, cognition, and ADL. Clinical signs of aspiration were present in 8.5% of the residents. Overall, 16.1% of residents exhibited reduced nutritional intake in the last three months, 0.7% received artificial nutrition by PEG, and 0.4% received nasogastric tube feeding. Reduced nutritional intake and artificial nutrition were more common in 2013 than in 2007 (OR_adj_: 1.23, 95% CI: 1.04–1.46, *P* = 0.013; and OR_adj_: 2.57, 95% CI: 1.21–5.44, *P* = 0.014, receptively). Chewing problems were observed among 11.4%, with no differences between years*.*

**Table 2:** Prevalence rates of swallowing ability, nutritional status, and chewing ability among nursing home residents between year 2007 and 2013

	Total	2007	2013	OR (95% CI)^a^	*P* value
Swallowing ability					
Oropharyngeal dysphagia^b^	712/4770 (14.9)	444/2740 (16.2)	268/2030 (13.2)	0.79 (0.66–0.95)	0.010
Clinical signs of aspiration^c^	403/4737 (8.5)	254/2720 (9.3)	149/2017 (7.4)	0.76 (0.61–0.96)	0.020
Nutritional status					
Reduced nutrition intake^d^	767/4752 (16.1)	417/2728 (15.3)	350/2024 (17.3)	1.23 (1.04–1.46)	0.013
Artificial nutrition				2.57 (1.21–5.44)	0.014
PEG tube	33/4590 (0.7)	12/2658 (0.5)	21/1932 (1.1)	3.00 (1.34–6.75)	0.008
Nasogastric tube	17/4611 (0.4)	6/2676 (0.2)	11/1935 (0.6)	3.34 (1.02–10.90)	0.046
Chewing ability					
Good	3118/4875 (64.0)	1775/2782 (63.8)	1343/2093 (64.2)	1.00 (0.87–1.15)	1.000
Quite good	1199/4875 (24.6)	686/2782 (24.7)	513/2093 (24.5)	1.03 (0.89–1.17)	0.680
Poor	558/4875 (11.4)	321/2782 (11.5)	237/2093 (11.3)	1.08 (0.88–1.32)	0.475

Data are given as *n* (%)

*PEG* percutaneous endoscopic gastrostomy, *OR* odds ratio, *CI* confidence interval

^a^Adjusted for age, sex, cognition, and activity of daily living

^b^Defined as difficulty in swallowing or showing clinical signs of aspiration during a meal within the last month as observed by a caregiver

^c^Defined as misdirected swallowing during a meal within the last month as observed by a caregiver

^d^Refers to reduced nutritional intake the last 3 months

### Associations with oropharyngeal dysphagia

Table 3 presents the associations between oropharyngeal dysphagia and other factors. Oropharyngeal dysphagia was significantly associated with cognitive impairment (*P* = 0.014), greater ADL dependency (*P* < 0.001), poor chewing ability (*P* < 0.001), reduced nutritional intake the last the three months (*P* < 0.001), and artificial nutrition (PEG: *P* < 0.001; nasogastric tube: *P* < 0.001). However, oropharyngeal dysphagia was not associated with age (*P* = 0.161), sex (*P* = 0.981) or polymedication (*P* = 0.269).

**Table 3:** Association between oropharyngeal dysphagia and health-related factors among residents in nursing homes in 2007 and 2013

Characteristic	Classification	Dysphagia *n* = 709	No Dysphagia *n* = 4042	*P* value
Age	(years, mean ± SD)	84.3 ± 7.1	84.8 ± 6.9	0.161^a^
Sex	Men	227 (32.0)	1296 (32.1)	0.981^b^
	Women	482 (68.0)	2746 (67.9)	
Cognition impairment	(0–27, mean ± SD)	12.7 ± 8.9	16.8 ± 8.4	0.014^a^
ADL	(4–24, mean ± SD)	10.4 ± 5.4	16.4 ± 6.0	< 0.001^a^
Depression	No	259 (37.7)	1741 (44.0)	0.003^b^
	Quite	309 (44.9)	1690 (42.8)	
	Very	95 (13.8)	438 (11.1)	
	Severe	25 (3.6)	85 (2.1)	
Polymedication^c^	No	444 (65.2)	2594 (67.4)	0.269^b^
	Yes	237 (34.8)	1257 (32.6)	
Nutritional status				
Reduced nutrition intake^d^	No	470 (69.6)	3435 (86.5)	< 0.001^b^
	Yes	205 (30.4)	538 (13.5)	
PEG	No	642 (96.5)	3810 (99.7)	< 0.001^b^
	Yes	23 (3.4)	10 (0.3)	
Nasogastric tube	No	651 (97.9)	3836 (99.9)	< 0.001^b^
	Yes	14 (2.1)	3 (0.01)	
Chewing ability	Good	130 (18.7)	2878 (71.8)	< 0.001^b^
	Quite good	279 (40.1)	880 (21.9)	
	Poor	287 (41.2)	252 (6.3)	

Data are given as *n* (%). Regarding missing data, the prevalence is based on complete cases for the specific variable in each analysis

*PEG* percutaneous endoscopic gastrostomy

^a^Independent t-test

^b^Chi-squared test

^c^Defined as regularly taking 5 or more different drugs

^d^Reduced nutritional intake in the last three months

**Fig. 2:**
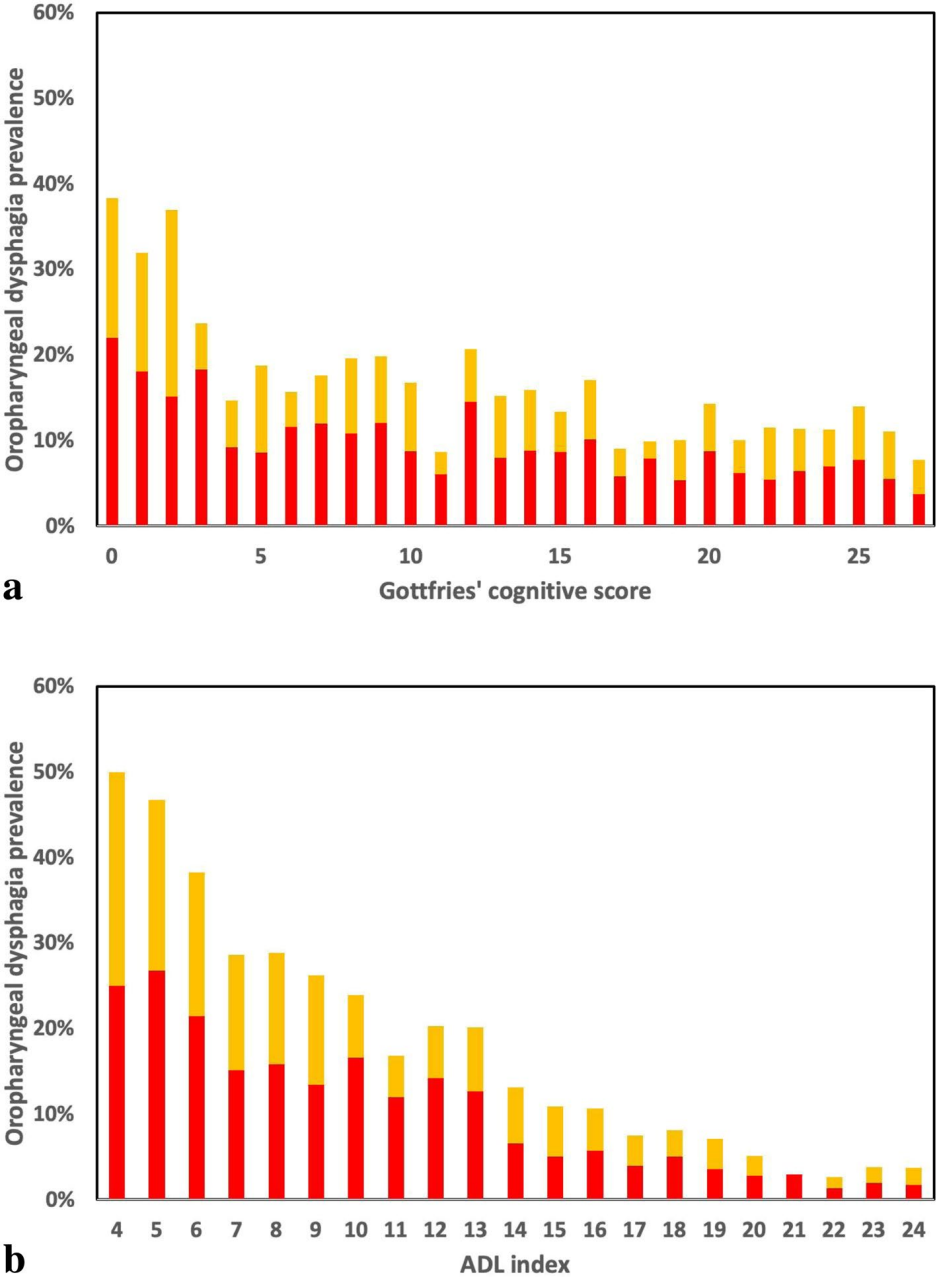
**a** Histogram of the prevalence of oropharyngeal dysphagia among nursing home residents (*n* = 4657) according to cognition level in year 2007 and 2013. The stacks represent residents with oropharyngeal dysphagia and are divided into residents that have shown clinical signs of aspiration (red) and swallowing difficulties only (yellow). **b** Histogram of the prevalence of oropharyngeal dysphagia among nursing home residents (*n* = 4539) according to the activity of daily living index in year 2007 and 2013. The stacks represent residents with oropharyngeal dysphagia and are divided into residents that have shown clinical signs of aspiration (red) and swallowing difficulties only (yellow)

Figure 2a shows the association between oropharyngeal dysphagia and cognition, according to the Gottfries’ cognitive scale. The figure illustrates a non-linear association between oropharyngeal dysphagia and cognition, with higher prevalence of oropharyngeal dysphagia observed with reduced cognition, especially among individuals with very low Gottfries’ scores (0–2) indicating very severe cognitive impairment. A similar but linear pattern is shown between oropharyngeal dysphagia and ADL, with a higher prevalence of oropharyngeal dysphagia observed with reduced ADL independence (Fig. 2b).

### Associated factors (potential predictors) of oropharyngeal dysphagia

Table 4 shows the results from the adjusted model regarding associated factors of oropharyngeal dysphagia. In the adjusted model, very low cognitive function, according to the dichotomized Gottfries’ cognitive scale (0–2 vs. > 2), indicated 1.56 times higher odds of having dysphagia (OR: 1.56, 95% CI: 1.14–2.12, *P* = 0.005). On the other hand, the Gottfries’ cognitive scale as a continuous variable (0–27) indicated that higher risk of oropharyngeal dysphagia was associated with increased cognition (OR: 1.05, 95% CI: 1.04–1.07, *P* < 0.001). Furthermore, the risk of having oropharyngeal dysphagia was 0.87 times lower with increased ADL independency (OR: 0.81, 95% CI: 0.79–0.83, *P* < 0.001).

**Table 4:** Associated factors (potential predictors) of oropharyngeal dysphagia among nursing home residents in year 2007 and 2013

Variables	OR (95% confidence interval)	
	Adjusted^a^	*P* value
Age	1.01 (0.99–1.02)	0.380
Sex (ref. men)	1.11 (0.91–1.34)	0.314
Year (ref. 2007)	0.80 (0.67–0.96)	0.017
Cognition (Gottfries’ continuous 0–27)	1.05 (1.04–1.07)	< 0.001
Cognition (Gottfries’ 0–2 *vs* > 2) (ref. > 2)	1.56 (1.14–2.12)	0.005
ADL (4–24)	0.81 (0.79–0.83)	< 0.001

*OR* odds ratio, *ADL* activity of daily living

For the logistic regression models (univariable and multivariable), the reference categories were: men sex, year 2007, and Gottfries’ cognition score > 2. Cognition was modelled using one continuous variable and one dichotomized variable, allowing a non-linear association between dysphagia and cognition

^a^Multivariable model

### Associated factors (potential consequences) of oropharyngeal dysphagia

Table 5 presents the results of regression models for the association between oropharyngeal dysphagia and artificial nutrition, reduced nutrition intake, and depression (adjusted for age, sex, year, cognition, and ADL). The models showed that presence of oropharyngeal dysphagia was associated with 10.89 times higher risk of clinical signs of aspiration (_adj_OR: 10.89, 95% CI: 8.40–14.12, *P* < 0.001), 6.33 times higher odds of requiring artificial nutrition (_adj_OR: 6.33, 95% CI: 2.73–14.71, *P* < 0.001), and 1.84 times higher odds of reduced nutritional intake (_adj_OR: 1.84, 95% CI: 1.49–2.27, *P* < 0.001). We found no association between oropharyngeal dysphagia and depression (_adj_OR: 1.19, 95% CI: 0.99–1.00, *P* = 0.063).

**Table 5:** Regression models analyzing associated factors (potential consequences) of oropharyngeal dysphagia among nursing home residents in year 2007 and 2013

Dependent variables	OR (95% confidence interval)	
	Adjusted odds ratio for individuals with oropharyngeal dysphagia^a^	*P* value
Clinical signs of aspiration (ref. no)^bc^	10.89 (8.40–14.12)	< 0.001
Reduced nutritional intake^d^(ref. no)	1.84 (1.49–2.27)	< 0.001
Artificial nutrition^e^ (ref. no)	6.33 (2.73–14.71)	< 0.001
Depression (ref. no)	1.19 (0.99–1.00)	0.063

*OR* odds ratio

For the logistic regression models the reference categories were: no clinical signs of aspiration, normal nutritional intake, no artificial nutrition, and no indication of severe depression

^a^Multivariable models were adjusted for the following confounders: age, sex, year, activity of daily living, and cognition

^b^The model was analyzed with clinical signs of aspiration separated from the overall definition of oropharyngeal dysphagia (the resident has shown difficulty in swallowing and/or clinical signs of aspiration)

^c^Refers to misdirected swallowing

^d^Indicates reduced nutritional intake in the last three months

^e^Defined as either percutaneous endoscopic gastrostomy tube or nasogastric tube feeding

## Discussion

In this cross-sectional survey study, we found that oropharyngeal dysphagia, as reported by caregivers, was present in approximately 15% of the nursing home residents in northern Sweden. An adjusted analysis identified cognitive impairment and ADL as associated factors with oropharyngeal dysphagia. A strength of this study is that cognitive and functional assessments were performed within an extensive and inclusive nursing home study sample, enabling the analysis of oropharyngeal dysphagia in association to both mild and severe cognitive impairment. Below, we discuss specific considerations regarding the prevalence of oropharyngeal dysphagia, nutritional status, and chewing ability, and associated factors (potential predictors and consequences) of oropharyngeal dysphagia.

In the present study, oropharyngeal dysphagia was observed in 14.9% of nursing home residents, and clinical signs of aspiration in 8.5%. This prevalence rate is similar to the previously reported rates of oropharyngeal dysphagia among nursing home residents, as assessed by caregivers. Huppertz et al. [13] found that oropharyngeal dysphagia was present in approximately 12% of the nursing home residents when assessed by their caregivers’ answers to a polar question (if a resident had dysphagia or not). Streicher et al. [12] found that caregiver-reported oropharyngeal dysphagia was present among 13.4% of 23,549 residents of nursing homes from 19 countries. In the study by Peladic et al. [14], 12.8% of the nursing home residents had oropharyngeal dysphagia, and 16% received artificial nutrition. Our present findings support the knowledge that oropharyngeal dysphagia is an existing problem among nursing home residents.

Although the sensitivity and specificity might be lower when using a proxy method, the advantage of the proxy method in the current study is that it allows the inclusion of all residents living in nursing homes in northern Sweden, even those with severe major neurocognitive disorders. One could speculate that the prevalence might have been higher if the residents’ swallowing ability had been objectively assessed using a clinical or instrumental swallowing assessment. Other studies using validated assessments tools has shown higher prevalence rate of oropharyngeal dysphagia in nursing homes [1, 11]. Early identification of residents at risk of oropharyngeal dysphagia is essential for effective management to minimize malnutrition, dehydration, and pneumonia. To prevent adverse outcomes, it could be advocated that a simple screening tool for oropharyngeal dysphagia should be implemented as part of daily care in nursing homes in Sweden.

In the current study, reduced nutritional status was found among 16% of the residents. This result is in line with the findings of Nordenram et al. [22], which showed reduced food intake in 14% of nursing home residents, when assessed by the ward staff. An even higher prevalence of malnutrition has been observed among nursing home residents when assessed using instruments, such as the Minimal Nutrition Assessment (MNA) [23–26]. Poor chewing ability was observed among 11.4% of residents in our study. This prevalence is lower than in other studies, where chewing problems have been observed in 23–26% of nursing home residents [12, 22]. The higher rate of chewing problems in other studies might be due to several factors. One is the method used, for example, if a dental examination by a dentist/dental hygienist with a valid tool was carried out or if the chewing ability was assessed by caregivers based on a question. The clinical distinction between impairment in mastication and difficulties in the oral phase of swallowing might also be challenging for a caregiver without training (or a validated assessment tool). Another factor that might impact the difference in the rate of chewing problems between studies is the advance in oral health care and treatment in recent years, especially in Sweden [27].

We also observed that the rates of oropharyngeal dysphagia and reduced nutritional status differed between the two survey years. Oropharyngeal dysphagia was somewhat less present among nursing home residents in 2013 compared to in 2007 (OR: 0.79. While individuals with dysphagia had 1.84 times higher odds of having decreased nutritional intake, the odds of having either artificial nutrition or reduced nutritional intake were still higher in 2013. These findings might illustrate the complexity of the associations between these and other contributing factors. Chewing ability did not differ between the two survey years.

In our study, the most prominent associated factors (and thus potential predictors) for oropharyngeal dysphagia were severe cognitive impairment and ADL capability. These findings are in line with those reported in other studies. Similar to in our study, Streicher et al. [12] found higher oropharyngeal dysphagia risk among nursing home residents with severe cognitive impairment and artificial nutrition, chewing problems. The relation between oropharyngeal dysphagia and cognition might be explained by the fact that severe cognitive impairment is associated with increased deterioration of the swallowing network in the brain, affecting the ability to safely and effectively swallow food, drinks, and medicine [1, 9, 28].

Although oropharyngeal dysphagia was more prevalent among residents with severe cognitive impairment, we also discovered that the risk of oropharyngeal dysphagia increased with increasing cognitive function. In a nursing home population, this seemingly surprising finding might be explained by the fact that oropharyngeal dysphagia is also common among people with other neurological diseases, such as stroke or Parkinson’s disease [1], or can be due to muscular weakness and sarcopenia [29], which are not necessarily associated with cognitive impairment. Notably, in Sweden, there is a high threshold in terms of disability for nursing home placement; therefore, all nursing home residents have cognitive or functional disability or very poor general health, and the prevalence of other disorders is likely very high among those with intact cognition. This high rate of other disorders might be related to oropharyngeal dysphagia and be more prominent in the slightly younger residents living in nursing homes in northern Sweden. However, this is only a hypothesis since we do not have any data regarding the residents’ medical records in the current study.

Similar to Streicher et al. [12] we found that chewing problems were associated with oropharyngeal dysphagia, but we did not include it in the adjusted model as it could be seen in as cofounding factor. Chewing problems might result from oropharyngeal dysphagia and diseases affecting the ability to chew [1], but may also be related to the number of teeth and occlusion areas [15] and as mentioned above the clinical distinction between impaired mastication and oral dysphagia can be difficult to make without training or validated assessment tool.

In our study, age was not associated with the odds of having dysphagia. Interestingly, this effect of age on oropharyngeal dysphagia diverges from some of the literature among nursing home residents. In line with the current study, Streicher et al. [12] and Peldaic et al. [14] did not find that age was an independent risk factor for oropharyngeal dysphagia, whereas Park et al. [11] found that advanced age (75 years or older) was a risk factor associated with oropharyngeal dysphagia. These contradictory findings might illustrate that age, per se, does not cause oropharyngeal dysphagia, but that increased age is associated with an increased prevalence of age-related diseases that affect the swallowing process, such as major neurocognitive disorder and stroke [1]. However, the sample size and methodology used to diagnose oropharyngeal dysphagia differ between studies, making it difficult to compare the results [11, 12, 14].

ADL was also found to be a significant associated factor (and potential predictor) of oropharyngeal dysphagia in our study. We found that the odds of having oropharyngeal dysphagia was lower with increased ADL independence, i.e., the prevalence of dysphagia is higher with increased ADL dependency. This finding is not surprising considering that oropharyngeal dysphagia may be caused by neurologic diseases, including major neurocognitive disorder, and by muscular weakness and sarcopenia [1, 8, 29], which may be expected among Swedish nursing home residents. Our findings also align with other studies in which ADL dependency, especially severe dependency, has been identified as an associated factor for oropharyngeal dysphagia [9, 11, 12, 14].

Our findings support previous knowledge regarding associated factors seen as potential dysphagia-related consequences [1, 10]. We found that aspiration, artificial nutrition, and reduced nutritional intake were associated with oropharyngeal dysphagia. For example, compared to those without oropharyngeal dysphagia, residents with oropharyngeal dysphagia had 6.33 times higher odds of receiving artificial nutrition. These findings are not surprising since aspiration results from an unsafe swallow. Additionally, severe oropharyngeal dysphagia, carrying a high risk of aspiration, often results in the provision of artificial nutrition to ensure the necessary daily amount of calories and water to be well-nourished and hydrated. Finally, an unsafe or inefficient swallow may result in reduced daily nutritional intake, leading to malnutrition.

Our study illustrates that several health-related factors are associated factors of oropharyngeal dysphagia (severe cognitive impairment, ADL capability, reduced nutritional status, artificial nutrition, and clinical aspiration signs). Although no causal relationship can be established on a cross-sectional data, these associated factors with oropharyngeal dysphagia could be seen as potential predictors (severe cognition and functional status) or as its potential consequences (artificial nutrition, reduced nutritional status, and aspiration), and that these factors are common among nursing home residents. These findings highlight the need for routine assessment of swallowing, nutrition status, and oral health in all nursing home residents since these factors often co-exist, are independent risk factors, and appear to have a strong interrelationship in the older population.

This study has several limitations that must be acknowledged. First, swallowing ability was assessed using a proxy method based on a non-validated screening with questions. Although the caregivers have more contact with the residents and thus are most likely to observe clinical signs of oropharyngeal dysphagia, the caregivers had not received any training in identifying dysphagia. The prevalence rate of oropharyngeal dysphagia might thus have been higher if the caregivers had received training. A higher oropharyngeal dysphagia prevalence might also have been observed if an instrumental assessment with FEES or VFS had been applied. Second, nutritional status was not assessed using a valid tool, such as the MNA or the Minimal Eating Observation and Nutrition Form-II (MEONF-II) [30], which would have enabled accurate diagnosis and further management. Third, we did not include assessment of the number of teeth or occlusion areas, which are important factors associated with chewing and oropharyngeal dysphagia [15]. Fourth, some cases might be included in 2007 and 2013 due to the proxy method used where no data on personal ID is collected. However, the median survival time after nursing home placement is relatively short in Sweden, and therefore few residents have stayed longer than six years. This potential limitation is not judged to impact the current study's findings significantly. Fifth, it would have been interesting to investigate the association between medical diagnosis and oropharyngeal dysphagia, and to estimate and compare the mortality rates among residents with and without oropharyngeal dysphagia. Finally, to establish cause-effect relationships longitudinal studies are warranted, since this is not possible on a cross-sectional study. Future studies should also evaluate the implementation of a simple dysphagia screening tool among nursing home residents, for early detection to minimize its consequences.

## Conclusions

Oropharyngeal dysphagia was reported among approximately 15% of nursing home residents in northern Sweden. Associated health-related factors with oropharyngeal dysphagia included cognitive impairment and ADL capability. Furthermore, reduced nutritional status, artificial nutrition, and clinical aspiration signs were associated with oropharyngeal dysphagia. Implementing routine protocols in nursing homes may help detect oropharyngeal dysphagia and manage oropharyngeal dysphagia among residents.

## Data Availability

The datasets used and/or analyzed during the current study are available from the corresponding author on reasonable request.

## Abbreviations

ADL: Activity of daily living
CI: Confidence interval
MNA: Minimal Nutrition Assessment
MEONF-II: Minimal Eating Observation and Nutrition Form-II
MMSE: Mini-Mental State Examination
OR: Odds ratio
